# *Lacticaseibacillus rhamnosus* CRL 2244 secreted metabolites display killing and antibiotic synergistic activity against multi-drug resistant pathogens

**DOI:** 10.1371/journal.pone.0306273

**Published:** 2024-06-28

**Authors:** Cecilia Rodriguez, Dema Ramlaoui, Briea Gasca, Adiba Azis, Camila Leal, Christina Lopez, Vyanka Merzcord, Kirsten S. McManus, Jasmin Jo, Silvia I. Cazorla, Tomás Subils, Marisel R. Tuttobene, Nicholas T. Salzameda, Robert A. Bonomo, Luis A. Actis, Raúl Raya, María Soledad Ramirez

**Affiliations:** 1 Centro de Referencia para Lactobacilos (CERELA), CONICET, Tucumán, Argentina; 2 Center for Applied Biotechnology Studies, Department of Biological Science, College of Natural Sciences and Mathematics, California State University Fullerton (CSUF) Fullerton, CA, United States of America; 3 Department of Chemistry and Biochemistry, College of Natural Science and Mathematics, CSUF, Fullerton, CA, United States of America; 4 Instituto de Procesos Biotecnológicos y Químicos de Rosario (IPROBYQ, CONICET-UNR), Rosario, Argentina; 5 Instituto de Biología Molecular y Celular de Rosario (IBR, CONICET-UNR), Rosario, Argentina; 6 Research Service and GRECC, Louis Stokes Cleveland Department of Veterans Affairs Medical Center, Cleveland, OH, United States of America; 7 Departments of Medicine, Pharmacology, Molecular Biology and Microbiology, Biochemistry, Proteomics and Bioinformatics, Case Western Reserve University School of Medicine, Cleveland, OH, United States of America; 8 CWRU-Cleveland VAMC Center for Antimicrobial Resistance and Epidemiology (Case VA CARES), Cleveland, OH, United States of America; 9 Department of Microbiology, Miami University, Oxford, OH, United States of America; The University of Lahore, PAKISTAN

## Abstract

A growing increase in the number of serious infections caused by multidrug resistant bacteria (MDR) is challenging our society. Despite efforts to discover novel therapeutic options, few antibiotics targeting MDR have been approved by the Food and Drug Administration (FDA). Lactic acid bacteria have emerged as a promising therapeutic alternative due to their demonstrated ability to combat MDR pathogens *in vitro*. Our previous co-culture studies showed *Lacticaseibacillus rhamnosus* CRL 2244 as having a potent killing effect against carbapenem-resistant *Acinetobacter baumannii* (CRAB) strains. Here we report that cell-free conditioned media (CFCM) samples obtained from *Lcb*. *rhamnosus* CRL 2244 cultures incubated at different times display antimicrobial activity against 43 different pathogens, including CRAB, methicillin-resistant *Staphylococcus aureus* (MRSA) and carbapenemase *Klebsiella pneumoniae* (KPC)-positive strains. Furthermore, transwell and ultrafiltration analyses together with physical and chemical/biochemical tests showed that *Lcb*. *rhamnosus* CRL 2244 secretes a <3 kDa metabolite(s) whose antimicrobial activity is not significantly impaired by mild changes in pH, temperature and various enzymatic treatments. Furthermore, sensitivity and time-kill assays showed that the bactericidal activity of the *Lcb*. *rhamnosus* CRL 2244 metabolite(s) enhances the activity of some current FDA approved antibiotics. We hypothesize that this observation could be due to the effects of *Lcb*. *rhamnosus* CRL 2244 metabolite(s) on cell morphology and the enhanced transcriptional expression of genes coding for the phenylacetate (PAA) and histidine catabolic Hut pathways, metal acquisition and biofilm formation, all of which are associated with bacterial virulence. Interestingly, the extracellular presence of *Lcb*. *rhamnosus* CRL 2244 induced the transcription of the gene coding for the CidA/LgrA protein, which is involved in programmed cell death in some bacteria. Overall, the findings presented in this report underscore the promising potential of the compound(s) released by *Lcb*. *rhamnosus* CRL2244 as an alternative and/or complementary option to treat infections caused by *A*. *baumannii* as well as other MDR bacterial pathogens.

## Introduction

Antibiotic resistance in bacteria constitutes a prominent and escalating menace to public health, jeopardizing healthcare systems, food security, and global progress. In recent years a significant surge in infections caused by highly antibiotic-resistant bacteria has been reported, leading to a substantial increase in healthcare expenses and necessitating the development of novel therapeutic agents [1, 2]. In this context, carbapenem-resistant *Acinetobacter baumannii* (CRAB) has earned a critical priority status from both the World Health Organization (WHO) and the Centers for Disease Control and Prevention (CDC) [3, 4]. The global prevalence of *A*. *baumannii* strains resistant to multiple classes of antibiotics underscores the pressing demand for novel antimicrobial therapies. Despite concerted efforts by various research groups and pharmaceutical companies during the past decade, the approval of new drugs tailored to combat *A*. *baumannii* has been limited [5–7]. Presently, only two drugs, cefiderocol and sulbactam-durlobactam, have secured approval from the U.S. Food and Drug Administration (FDA) for treating *A*. *baumannii* infections [8, 9].

The lack of significant progress in the development of novel and efficacious antibiotics emphasizes the urgency of exploring innovative methods to address CRAB infections. Current research is directed toward therapeutic alternatives, such as the application of lactic acid bacteria (LAB) to treat bacterial infections [10–13]. The growing interest in LAB can be attributed to their Generally Regarded as Safe (GRAS) status, intrinsic antimicrobial attributes, and the myriad of health advantages they provide to the human host, particularly in terms of enhancing the equilibrium of the intestinal microbiota [14]. Our group has recently reported that the *Lacticaseibacillus rhamnosus* CRL 2244 strain significantly inhibits the growth of both susceptible and CRAB strains of *A*. *baumannii* [15]. We also observed changes at both the transcriptomic level as well as in *A*. *baumannii* morphology when it was co-cultured in the presence of *Lcb rhamnosus* CRL 2244 [15].

Based on these prior observations, the aim of this study is to investigate whether *Lcb*. *rhamnosus* CRL 2244 secretes substances capable of triggering or contributing to the death of CRAB cells. To achieve this goal, we isolated cell-free conditioned medium (CFCM) from cultures of *Lcb*. *rhamnosus* CRL 2244 and performed a comprehensive analysis, including the evaluation of its antimicrobial activity against various CRAB strains and other multidrug resistant (MDR) pathogens. In addition, we also characterized some of its physicochemical properties, explored potential synergistic effects with other antibiotics, and the determined of how *A*. *baumannii* responds to CFCM, including its growth rate and the differential expression of virulence-related genes.

## Materials and methods

### Bacterial strains and culture conditions

The *Lcb*. *rhamnosus* CRL 2244 strain, which displays antimicrobial activity against *A*. *baumannii* [15], was used to prepare cell-free conditioning medium (CFCM) after two subcultures at 37°C in Man, Rogosa, and Sharpe (MRS) broth (Oxoid, Basingstoke, Hampshire, United Kingdom). Three *A*. *baumannii* model strains, the A118 antibiotic-susceptible strain [16, 17] and the CRAB AB5075 (*bla*_OXA-23_ and *bla*_OXA-51_) [18] and AMA3[19] clinical isolates were used in this work. In addition, a total of 40 strains, including MDR isolates (n = 34), and susceptible strains (n = 6) were used for the spot-on-the-lawn assays to test antimicrobial activity (Table 1). The strains were grown on Cystine-Lactose-Electrolyte-Deficient (CLED) medium (Beckton Dickinson, Franklin Lakes, NJ, USA) overnight at 37°C and used within 24 h.

**Table 1:** Antimicrobial activity of *Lacticaseibacillus rhamnosus* CRL 2244 CFCM against multidrug pathogens tested by the spot-on-the-lawn method.

Strains	IDH (mm)				Phenotype	Key resistance mechanisms^a^
	48 h	96 h	120 h			
*Acinetobacter baumannii*						
AMA2	20	15		10	MDR / CR	*bla*_NDM-1_
AMA3	16	20		20	MDR / CR	*bla*_NDM-1_, *bla*_PER-7_
AMA5	14	25		25	MDR / CR	*bla*_NDM-1_
AMA9	0	17		0	MDR / CR	*bla*_NDM-1_
AMA10	0	19		20	MDR / CR	*bla*_NDM-1_
AMA14	0	17		24	MDR / CR	*bla*_NDM-1_, *bla*_PER-7_
AMA16	21	20		14	MDR / CR	*bla*_NDM-1_, *bla*_PER-7_
AMA18	0	15		0	MDR / CR	*bla*_NDM-1_, *bla*_PER-7_
AMA22	0	17		21	MDR / CR	*bla*_NDM-1_
AMA30	17	19		21	MDR / CR	*bla*_NDM-1_, *bla*_PER-7_
AMA31	20	23		15	MDR / CR	*bla*_NDM-1_
AMA33	0	17		21	MDR / CR	*bla*_NDM-1_
AMA40	22	17		22	MDR / CR	*bla*_NDM-1_
AMA43	24	17		25	MDR / CR	*bla*_NDM-1_
AMA116	20	20		18	MDR / CR	*bla*_OXA-23_
AMA122	20	25		21	MDR / CR	*bla*_NDM-1_
AMA166	21	20		25	MDR / CR	*bla*_OXA-23_
AMANO	0	17		0	MDR / CR	*bla*_NDM-1_, *bla*_OXA-23_
AB0057	15	23		0	MDR / CR	*bla*_OXA-23_, *bla*_TEM-1_
AB5075	8	20		20	MDR / CR	*bla*_OXA-23_, *bla*_OXA-51_, *bla*_GES-14_
A118	11	18		16	S	
AB ATCC 17978	0	17		0	S	
*Escherichia coli*						
ECO 4	10	12		11	MDR	*aadA1*, *Inti*1
ECO 16	13	15		13	MDR	*aadA1*, *Inti*1
ECO 7499	15	19		20	MDR / CR	*bla*_KPC-2_
ECO PNDM5	16	21		23	MDR / CR	*bla*_NDM-1_
ECO ATCC 25922	20	21		20	S	
*Serratia marcescens*						
SmP9	12	12		12	MDR / CR	*bla*_KPC-2_, *bla*_CTX-M-2_
SmP7	11	10		10	MDR / CR	*bla*_KPC-2_, *bla*_CTX-M-2_, *bla*_SHV-2_
Sm 371	16	20		13	MDR / CR	*bla*_KPC-2_, *bla*_CTX-M-2_
Sm 909	14	13		11	MDR	*dfrA1*, *ant*(*3*”)*-Ia*, *ant*(*2*")*-Ia*, *aac*(*3*)*-Ia*
Sm 55	13	13		10	MDR / CR	*bla*_KPC-2_
*Klebsiella pneumoniae*						
KPN 1224	0	11		9	MDR / CR	*bla*_KPC-2_
KPN 25979	0	11		18	MDR / CR	*bla*_KPC-2_, *bla*_NDM-5_, *bla*_CTX-M-2_
KPN RB_NDM5	0	16		19	MDR / CR	*bla*_NDM-5_
*Salmonella*						
*S*. Schwarzengrund 1024	11	11		13	MDR / CR	*bla*_KPC-2_
*Staphylococcus aureus*						
SAU 96	17	18		15	MDR	*mecA*
USA 300	22	20		24	MDR	*mecA*
SAU RN4220	9	12		12	S	
SAU ATCC 25923	17	20		20	S	
*Providencia stuartii*						
*PS* 848	18	19		21	MDR / CR	*bla*_NDM-1_, *bla*_TEM-1_
*Staphylococcus epidermidis*						
ATCC 25933	22	21		16	S	
*Enterococcus faecalis*						
ATCC 29212	0	0		0	S	
Response summary						
Range	24–0	25–0		25–0		
Mean	11.74	17.04		15.06		
IDH_50_	14	17		16		

IDH, inhibition diameter halos; MDR, multidrug resistance (resistant at least to three different antibiotics families); CR, carbapenem resistant; *S*. *aureus* USA300, methicillin-resistant (MRSA) model strain.

^a^key relevant mechanisms of resistance, such as carbapenemases among others, are mentioned. In the susceptible strain no relevant mechanism of resistant in stated.

### Preparation of *Lcb*. *rhamnosus* CRL 2244 CFCM and detection of antimicrobial activity by spot-on-the-lawn assays

The evaluation of the antimicrobial activity of *Lcb*. *rhamnosus* CRL 2244 CFCM samples was performed following the modified well diffusion method described by Halder *et al*. (2017) [20]. Briefly, *Lcb*. *rhamnosus* CRL 2244 MRS broth (2% v/v inoculum) was incubated at 37°C for 48 h, 96 h and 120 h and CFCM samples were prepared after centrifugation at 9,200 x*g* for 5 min at 4°C and filtering the supernatant with 0.22 μm filters (Millipore, Billerica, MA, USA). Bacterial strains were suspended in sterile physiological solution (0.85% NaCl, w/v) at a concentration of 0.5 McFarland units (1.5 x 10^8^ CFU/ml). These inocula were then seeded onto CLED agar plates. A total of 25 μl of CFCMs from different incubation times were applied to the surface of the plates. Following 24 h of incubation at 37°C, the diameters of the growth inhibition halos (IDH) were measured and classified as less active (IDH ≤ 10 mm), moderately active (IDH = 11–14 mm), and very active (IDH ≥15 mm) [20]. The assays were performed using three independent biological samples.

### Transwell assays

Transwell inserts were used to confirm the diffusion of antimicrobial compounds secreted by *Lcb*. *rhamnosus* CRL 2244. Briefly, lysogeny broth (LB) cultures of A118 or AB5075 (1 x 10^7^ CFU/mL) were seeded onto transwell inserts (CELLTREAT Scientific Products, Pepperell, MA) with a permeable polyethylene terephthalate (PET) membrane with a pore size of 0.4 μm and exposed to undiluted and half-diluted overnight MRS broth cultures of *Lcb*. *rhamnosus* CRL 2244. After incubation at 37°C for 18 h, the cell density (OD_600_) and viability (CFU/mL) of *A*. *baumannii* cells exposed to *Lcb*. *rhamnosus* CRL 2244 cultures were determined by serial dilution and plating on CHROMagar™ *Acinetobacter* plates. The assays were performed using three independent biological samples.

### Preliminary characterization of *Lcb*. *rhamnosus* CRL 2244 CFCM

The effect of trichloroacetic acid (TCA), pH, temperature, and enzymes on the antimicrobial activity of compound(s) secreted by *Lcb*. *rhamnosus* CRL 2244 were studied using 96-h CFCM samples. Briefly, CFCM samples were treated with 20% cold TCA for 1 h and then centrifuged at 9,200 x*g* for 10 min in a microcentrifuge. The precipitate was washed 3 times with an ice-cold solution of 0.01 M HCl/90% acetone and resuspended in 100 mM Tris-HCl, pH 8.5, 8 M urea. To evaluate the effects of pH, CFCM samples were adjusted to pH 5.5, 6.5 and 8.0 with a 4 N NaOH solution. The effect of temperature was examined by treating CFCM samples at 25°C, 37°C, 50°C, 75°C and 100°C for 30 min. CFCM samples were treated separately with enzymes including proteinase K, lipase, alpha-chymotrypsin, pepsin, and catalase (all from Sigma, St. Louis, MO, USA). The enzyme reactions were carried out at a final enzyme concentration of 0.05 mg/mL for 30 min for catalase and of 0.5 mg/mL and 4 h for all other tested enzymes. All samples were incubated at 37°C. Untreated CFCM samples (pH 3.9) were used as a control. The antimicrobial activity of untreated and treated CFCM samples was tested on the AB5075 strain using the spot-on-the-lawn method. Technical triplicates using three independent biological samples were used to collect experimental data.

### Antimicrobial chemical extraction assays

Precipitation of active compound(s): An equal volume of ice-cold acetone was added to CFCM and vigorously stirred for 20 min. The mixture was refrigerated at 4°C for 2 h and the resulting precipitate was collected by centrifugation 8,000 x*g* and decanting of the supernatant. The procedure was repeated two more times with the supernatant and the combined precipitates were dissolved in water and then evaluated for antimicrobial properties.

Extraction of active compounds(s): In a separatory funnel, CFCM was mixed with ethyl acetate (10% volume of the CFCM). The ethyl acetate layer was separated from the aqueous phase and collected. The original aqueous layer was extracted two more times with ethyl acetate. The combined ethyl acetate extracts were washed with a saturated NaCl aqueous solution, dried over magnesium sulfate, filtered, and evaporated to remove the ethyl acetate and obtain crude extracts containing molecules of interest. The crude extract was dissolved in DSMO and evaluated for biological activity. The antibacterial activity of all CFCM extracts against AB5075 and AMA3 was determined by the spot-on-the-lawn method, calculating the mass of the extracts that were resuspended in DMSO, diluted to 1% DMSO, and adjusted to specific concentrations. An organic solvent control was included on the plate. Technical triplicates using three independent biological samples were used to collect experimental data for each tested extract.

### Minimum inhibitory activity and synergy assay of CFCMs

The CFCM obtained from a 96-h *Lcb*. *rhamnosus* CRL 2244 culture was lyophilized, weighed, and resuspended in phosphate buffered saline (PBS) to determine the minimum inhibitory concentration (MIC) by the broth microdilution method and minimum bactericidal concentration (MBC) assays, which were done following CLSI standards (CLSI 2020), against the AB5075 and AMA3 CRAB strains, and the MRSA USA300 isolate.

The synergy assays were performed by adding lyophilized CFCM at sub-inhibitory concentration (4 mg/mL) to cation-adjusted Muller-Hinton agar (CAMHA) medium. The gradient diffusion method was used to determine the MIC values for the AB5075 and AMA3 CRAB strains, *E*. *coli* 7499 and MRSA USA 300 using commercially available strips (Liofilchem S.r.l., Italy). The CRAB strains were tested for their susceptibility to meropenem (MEM), imipenem (IMP), ceftazidime/avibactam (CZA), amikacin (AK) and cefiderocol (CFDC). MRSA was tested for susceptibility to cefoxitin (FOX), ampicillin (AMP), cefazolin (CFZ), gentamicin (CN), erythromycin (ERY), trimethoprim-sulfamethoxazole (SXT) and vancomycin (VAN). CAMHA plates without added lyophilized CFCM were used as control. For each antibiotic evaluated, the fractional inhibitory concentration (FIC) was calculated as follows:

$$FIC=\frac{MIC\;(antibiotic\;in\;the\;presence\;of\;CFCM)\;}{MIC\;(antibiotic\;alone)}$$

A FIC of ≤ 0.5 indicates synergism, > 0.5–1 indicates additive effects, ≥ 2–4 indifference, and > 4 is considered to be antagonism [21].Technical triplicates using three indepndent biological samples were used to collect experimental data obtained using each tested condition.

### Effect of CFCM on *A*. *baumannii* colony morphology

Phenotypic changes in macrocolonies of the AMA3 and AB5075 strains exposed to lyophilized CFCM were evaluated. Briefly, 5 μl of overnight cultures of bacteria exposed to 4 mg/mL of CFCM were inoculated onto 40 mL plates of cation-adjusted Müller Hinton agar (CAMHA) with 2% glycerol and supplemented with 40 μg/mL of Congo red (Sigma-Aldrich), which allows the detection of the poly-β-(1–6)-N-acetylglucosamine (PNAG) biofilm component. Cultures of AMA3 and AB5075 not exposed to CFCM were included as controls. After 24–48 h of incubation at 37°C, macrocolony changes were observed and registered.

### Time-killing assay of CFCM

The viability of *A*. *baumannii* AB5075 and AMA3 in the presence of lyophilized CFCM at MIC, 1 sub-MIC and 2 sub-MIC concentrations was assessed following the protocol of Balouiri *et al*. [22], with modifications. Briefly, tubes with LB broth inoculated with a bacterial suspension of 5 x 10^8^ CFU/mL and the different concentrations of CFCM were incubated at 37°C for 24 h. Cells not exposed to CFCM were included as controls. Aliquots taken at various time intervals were serially diluted and plated on LB agar to determine the CFU/mL values of each tested sample. Technical triplicates using three independent biological samples were used to collect experimental data.

### Scanning electronic microscopy (SEM)

From overnight cultures of *A*. *baumannii* on LB agar incubated at 37°C, one colony was resuspended in 100 μl of physiological solution and inoculated into 2 mL of LB broth supplemented with 4 mg/mL of lyophilized CFCM. *A*. *baumannii* cultured without exposure to lyophilized CFCM was included as a control. After overnight incubation at 37°C, the samples were centrifuged at 2,700 x*g* for 4 min, resuspended in Karnovsky fixative solution and processed for observation in the ZeissSupra 55VP Scanning Electron Microscope (Germany) belonging to the Centro Integral de Microscopía Electrónica (CIME-CONICET—Universidad Nacional de Tucumán, Argentina) as described before [15].

### RNA extraction and quantitative reverse transcription polymerase chain reaction (qRT-PCR) assays

*A*. *baumannii* AB5075 and AMA 3 cells were grown in the presence and absence of 4 mg/mL (sub-MIC concentration) of lyophilized CFCM for 18 h at 37°C. Total RNA extractions were carried out in triplicate for each condition using a commercial kit (Direct-zol RNA Kit, Zymo Research). DNase-treated RNA was used for cDNA synthesis using the iScriptTM Reverse Transcription Supermix kit (Bio-Rad, Hercules, CA, USA) following the manufacturer’s protocol. The cDNA concentrations were adjusted to 50 ng/μL, and qPCR was performed using the qPCRBIO SyGreen Blue Mix Lo-ROX following the manufacturer’s instructions (PCR Biosystems, Wayne, PA, USA). Each qPCR assay included at least three biological replicates of cDNA and run in triplicate utilizing the CFX96 TouchTM Real-Time PCR Detection System (Bio-Rad, Hercules, CA, USA). Data are presented as NRQ (Normalized relative quantities) calculated by the qBASE method [23, 24], using *recA* and *rpoB* genes as normalizers. Statistically significant differences were denoted with asterisks and determined through ANOVA followed by Tukey’s multiple comparison test (*P* < 0.05), employing GraphPad Prism (GraphPad Software, San Diego, CA, USA).

## Results

### Antimicrobial activity of CFCMs from *Lcb*. *rhamnosus* CRL 2244 against MDR pathogens

The antimicrobial activity of CFCMs obtained from *Lcb*. *rhamnosus* CRL 2244 cultures incubated for 48 h (CFCM-48), 96 h (CFCM-96), and 120 h (CFCM-120) was evaluated against a total of 43 pathogenic strains, 34 of which exhibited MDR and 31 also possessed carbapenemases coding genes (CR) (Table 1, S1 Fig). The CFCMs showed variable activity against the tested strains, with growth inhibitory halo diameters ranging from 0 to 24/25 mm. None of the tested CFCMs exhibited activity against *Enterococcus faecalis* ATCC 29212. The highest antimicrobial activity was achieved with CFCM-96, which was active against most of the tested strains (97.7%) with an inhibition diameter halo (IDH) mean of 16.95 mm and an IDH_50_ equal to 17 mm (Table 1, S1 Fig). CFCM-120 samples also showed antimicrobial activity, although less than CFCM-96 as evidenced by significantly smaller IDH (15.06 mm) and IDH_50_ (16 mm) means and a loss of activity against five of the tested strains. CFCM-48 demonstrated the lowest activity (IDH mean = 11.74 mm and IDH_50_ = 14 mm), and it failed to inhibit 11 of the tested strains (Table 1).

**Fig 1:**
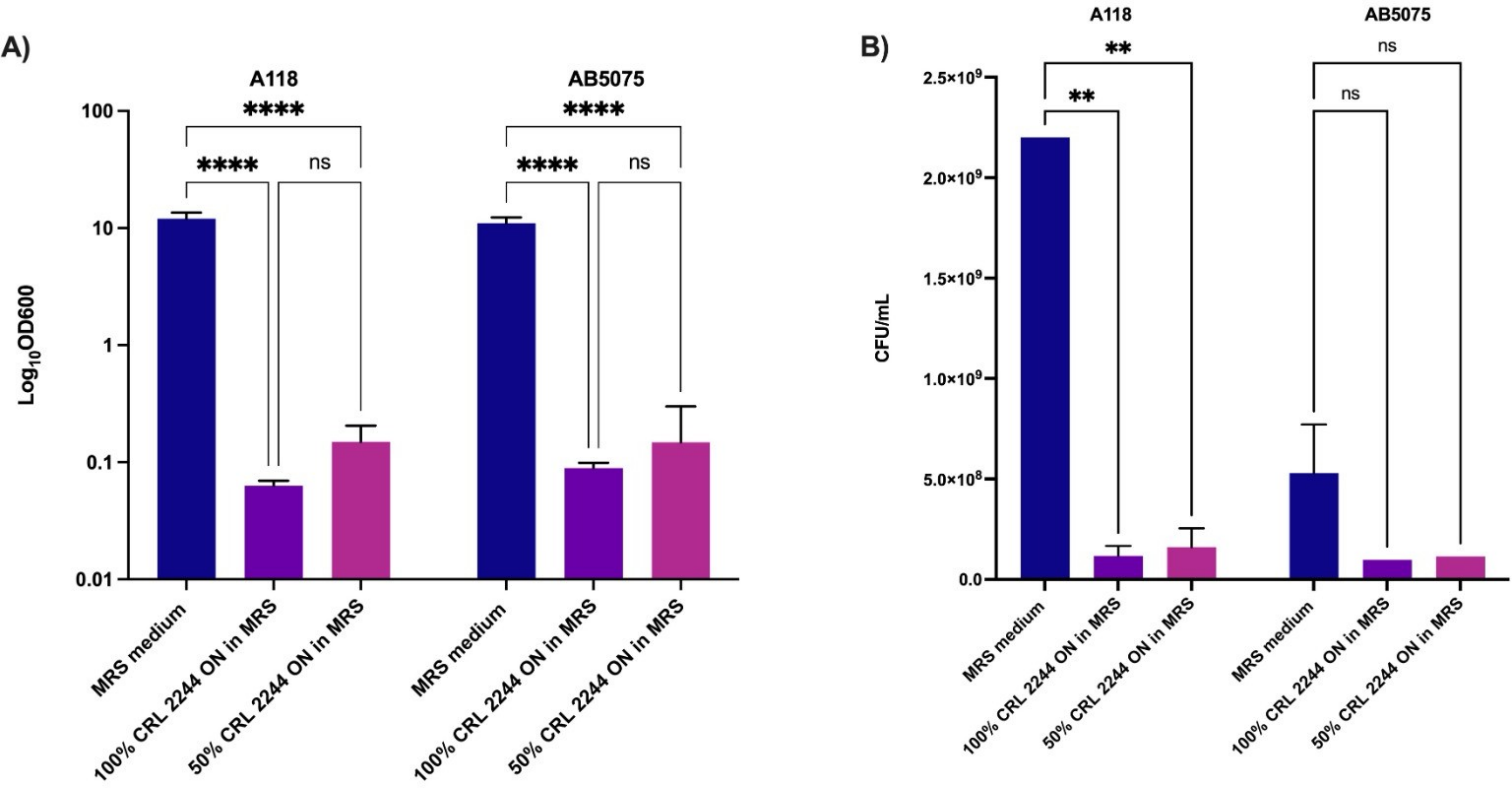
*Lcb*. *rhamnosus* CRL 2244 secretes compounds that alter *A*. *baumannii*. growth. A) Cell density (OD_600_), B) Surviving cells (CFU/mL). Transwell assays were performed using the *A*. *baumannii* clinical isolates A118 and AB5075. Bacteria were exposed to pure (100%) or diluted (50%) overnight *Lcb*. *rhamnosus* CRL 2244 cultures for 18 h at 37°C. Unexposed culture samples were used as controls. Technical triplicates using three independent biological samples were used to collect experimental data. Statistical significance (*P* < 0.05) was determined by two-way ANOVA followed by Tukey’s multiple comparison test. ****, *P* < 0.0001; ns, not significant. Error bars represents the standard deviation (SD).

Transwell assays confirmed that the antimicrobial activity of *Lcb*. *rhamnosus* CRL 2244 on strains AB5075 and A118 is due to the production of a compound secreted into the culture medium (Fig 1). The cell density of A118 and AB5075 broth cultures was drastically reduced (OD_600_ values near 0.1) after 18 h exposure to pure (100%) and diluted (50%) *Lcb*. *rhamnosus* CRL 2244 CFCM samples collected after overnight (ON) incubation (Fig 1A) when compared to the control group, which was not exposed to CFCM. In these experiments, the number of surviving cells for strain A118 was less than 1x10^-8^, while no cells were detected for strain AB5075 (Fig 1B), indicating the potent lethal activity of the antimicrobial compound present in the CFCM samples. These results suggest that *Lcb*. *rhamnosus* CRL 2244 secretes one or more soluble and diffusible compounds that significantly inhibit *A*. *baumannii* cell growth.

To start the characterization of the active component(s) secreted by *Lcb*. *rhamnosus* CRL 2244, the CFCM-96 was subjected to different treatments. CFCM samples treated with TCA 20% displayed similar antimicrobial effects when compared to untreated samples (Table 2). The antimicrobial activity of CFCM remained relatively stable in the mild acidic to basic pH tested range, as well as after heat treatment (Table 2). Similarly, treatment with a variety of enzymes (protease, peptidase, lipase, and oxidoreductase) did not significantly affect antibacterial activity. An IDH = 19 was obtained for pepsin and α-chymotrypsin and 20 mm for proteinase K, lipase and catalase (IDH = 20 mm for the CFCM-non treated control).

**Table 2:** Stability of the antimicrobial activity of *Lacticaseibacillus rhamnosus* CRL 2244 CFCM subjected to different treatments.

CFCM (IDH mm)												
**Strain**	Protein-free		pH				Temperature					
	TCA	Control	5.5	6.5	8.0	Control	25°	37°	50°	75°	100°	Control
**AB5075**	18	17	20	20	20	20	20	22	22	18	22	21

IDH, Inhibition diameter halo.

Control, CFCM non-treated, pH = 3.9 at room temperature.

The active compound(s) secreted by *Lcb*. *rhamnosus* CRL 2244 were extracted from the CFCM by precipitation with acetone and extraction with ethyl acetate. The compound(s) recovered from all extraction methods retained the growth inhibitory activity against AB5075, with IDH values similar to those observed for the crude CFCM sample (S1 Table). Furthermore, CFCM samples that underwent ultrafiltration using a 3-kDa molecular weight cutoff filter demonstrated antimicrobial effectiveness against the AB5075 and AMA3 CRAB strains comparable to that observed with untreated CFCM control. An IDH of 22 mm was obtained for both strains.

Taken together, these results indicate that the CFCM active component(s) is likely a secreted metabolite that is a non-peptide polar organic molecule soluble in organic solvents, smaller than 3 kDa in size, stable within a mild acidic-basic range, and relatively resistant to increased temperatures and various enzymes treatments.

### Effect of *Lcb*. *rhamnosus* CRL 2244 CFCM on antibiotic susceptibility and colony morphology

Lyophilized *Lcb*. *rhamnosus* CRL 2244 CFCM samples were dissolved in PBS to determine MIC and MBC activities. The lyophilized CFCM MIC value was 8 mg/mL for both CRAB tested strains (AB5075 and AMA3), while the MIC value for the MRSA USA 300 strain was one dilution higher (15 mg/mL). The MBC values for the CRAB strains were consistent with the MIC values, whereas MBC value for USA 300 was 62 mg/mL (2 doubling dilution higher).

To evaluate the synergy of the antimicrobial compound(s) secreted by *Lcb*. *rhamnosus* CRL 2244 with clinically relevant antibiotics, a sub-inhibitory concentration of the lyophilized CFCM (4 mg/ml) was tested. For the AB5075 and AMA3 strains, a 4- and 5-fold dilution decrease in the MIC of meropenem was seen for each strain, respectively (Table 3, S2 Fig). Furthermore, strain AB5075 exhibited a reduction of 1-fold dilution for imipenem, ceftazidime/avibactam, and cefiderocol (Table 3, S2 Fig). MIC changes were not observed when AMA3 was tested for ceftazime/avibactam and cefiderocol. Similarly, synergy was not detected when amikacin was tested using the same experimental conditions. The same amikacin MIC of 128 mg/L was obtained for AB5075 with and without lyophilized CFCM. In addition, synergy for meropenem was also evaluated for *E*. *coli* 7499 (KPC producer) and, as expected, a synergistic effect was observed (Table 3, S2 Fig). The MRSA USA 300 MIC values for cefoxitin (MIC: 16 mg/L) and ampicillin (MIC: 1 mg/L) slightly decreased when the medium was supplemented with lyophilized CFCM (Table 3, S2 Fig). This strain also showed one-fold (MIC: 8 mg/L) and two-fold (MIC: 0.25 mg/L) dilution decreases for cefoxitin and ampicillin, respectively. In addition, the MRSA USA 300 strain exhibited a 2-dilution reduction for vancomycin in the presence of lyophilized CFCM (Table 3, S2 Fig).

**Table 3:** Synergy between *Lacticaseibacillus rhamnosus* CRL 2244 CFCM and antibiotics on multidrug resistant strains.

Strains	MIC values (mg/L)			
AB5075	CAMHA	CAMHA + 4 mg/mL CFCM	FIC	Effects
MEM	32	2	0.06	synergism
IMP	8	4	0.50	synergism
CAZ/AVI	128	64	0.50	synergism
FDC	0.5	0.25	0.50	synergism
AMA3	CAMHA	CAMHA + 4 mg/mL CFCM	FIC	Effects
MEM	64	0.94	0.01	synergism
IMP	>256	96	0.37	synergism
CAZ/AVI	>256	>256	1	additive
FDC	3	3	1	additive
*E*. *coli* 7499	CAMHA	CAMHA + 4 mg/mL CFCM	FIC	Effects
MEM	1	0.19	0.19	synergism
IMP	4	8	2	indifference
CAZ/AVI	0.94	0.94	1	additive
FDC	0.19	0.19	1	additive
*S*. *aureus* USA300	CAMHA	CAMHA + 4 mg/mL CFCM	FIC	Effects
AMP	1	0.25	0.25	synergism
FOX	16	8	0.50	synergism
CFZ	1	1	1	additive
CN	0.125	0.5	4	indifference
ERY	0.5	0.5	1	additive
SXT	0.064	0.064	1	additive
VAN	1	0.25	0.25	synergism

CAMHA, Cation-adjusted Mueller-Hinton Agar; MEM, meropenem; IMP, imipenem; CAZ/AVI, ceftazidime-avibactam; FDC, cefiderocol; AMP, ampicillin; FOX, cefoxitin; CFZ, cefazolin; CN, gentamicin; ERY, erythromycin; SXT, trimethoprim-sulfamethoxazole; VAN, vancomycin.

In addition, the ability of the CRAB strains to produce poly-N-acetylglucosamine (PNAG), which is a major component of the biofilm extracellular matrix, in the presence or absence of a sub-inhibitory concentrations of CFCM was examined using the Congo red dye assay [25]. A notable decrease in the roughness of the edges and a slight lightening in the red color tone of the colonies were observed when these two CRAB strains were exposed to the lyophilized CFCM (S3 Fig).

Taken together, these findings suggest that CFCM acts synergistically with β-lactam antibiotics, increasing the susceptibility of MDR strains to these antibiotics. In addition, CFCM induces alterations in the morphological phenotype of macrocolonies that could influence biofilm formation. These responses would influence changes in antibiotic resistance and affect *A*. *baumannii* virulence.

### Killing activity of *Lcb*. *rhamnosus* CRL 2244 CFCM on *A*. *baumannii*

**Fig 2:**
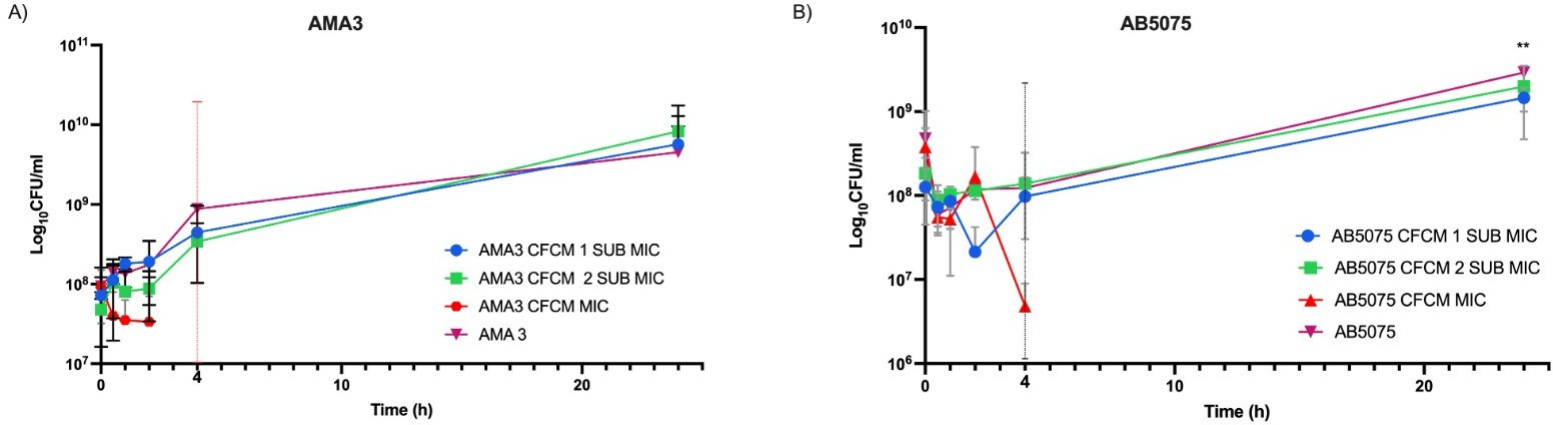
*Lcb*. *rhamnosus* CRL 2244 CFCM killing activity against *A*. *baumannii*. The clinical isolates AMA3 (A) and AB5075 (B) were incubated at 37°C for 24 h in presence of lyophilized CFCM at MIC, 1 sub-MIC and 2 sub-MIC concentrations. CFU/mL were determined at different incubation times for a period of 24 h. All assays were carried out in technical triplicates. Statistical significance (*P* < 0.05) was determined by two-way ANOVA followed by Tukey’s multiple comparison test. * *P* < 0.05, ** *P* < 0.01, *** *P* < 0.001 and **** *P* < 0.0001.

Time-kill assays were used to evaluate the bactericidal or bacteriostatic activity of lyophilized CFCM on AB5075 and AMA3 (Fig 2). At MIC concentrations, both CRAB strains showed a clear decrease in CFU/mL after short exposure to *Lcb*. *rhamnosus* CRL 2244 CFCM, and no cells were recovered after 24 h incubation. Concentrations below MIC affected the growth of AB5075 and AMA3 during the first 2 h, with slightly lower CFU values compared to the control. However, after 4 h, both strains showed an increase in CFU until reaching values similar to the control after 24 h incubation (Fig 2).

These results indicate that CFCM exhibits bactericidal behavior at concentrations equivalent to MIC. Whereas, at sub-MIC concentrations, CFCM induces less growth during the initial 8 hours of incubation.

### *Lcb*. *rhamnosus* CRL 2244 CFCM exerts cell morphological changes

**Fig 3:**
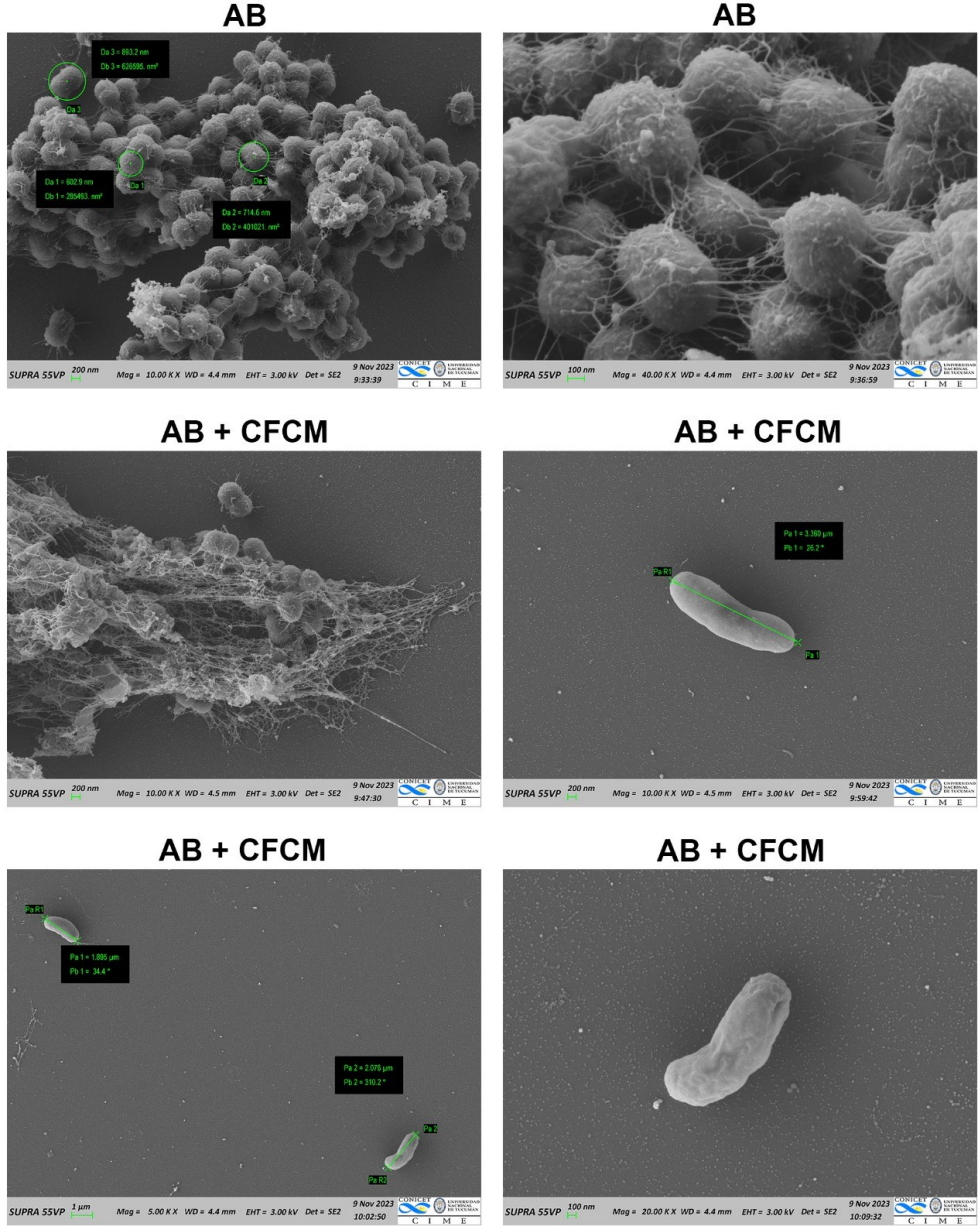
Scanning electron microscopy of *A*. *baumannii* cells cultured in the presence of *Lcb*. *rhamnosus* CRL 2244 lyophilized CFCM and resuspended in PBS. Micrographs were captured at a magnification of x5.000, ×10.000, x20.000 or x40.000. Bars, 1 μm, 100 nm or 200 nm.

Scanning electron microscopy (SEM) was performed to assess the impact of lyophilized CFCM at a sub-MIC concentration on the morphology of *A*. *baumannii* cells (Fig 3). Microscopic images revealed a significant reduction in cell density and extracellular matrix when AB5075 cells were treated with lyophilized CFCM. Cells were observed as dispersed in single or paired formations, contrasting with the homogeneously distributed and biofilm-immersed control cells (Fig 3). Furthermore, exposure to lyophilized CFCM induced significant changes in cell length, with exposed cells being up to 5 times longer than non-exposed control cells (3,500 nm *vs* 700 nm). In addition, the cell surface of exposed cells displayed a smother texture when compared to the roughness observed in control cells (Fig 3).

Taken together, these results suggest that CFCM could influence the ability of AB5075 cells to form multicellular aggregates resembling biofilm structures on a solid surface and alter cell morphology.

### Transcriptional response of *A*. *baumannii* to the presence of *Lcb*. *rhamnosus* CRL 2244 CFCM

**Fig 4:**
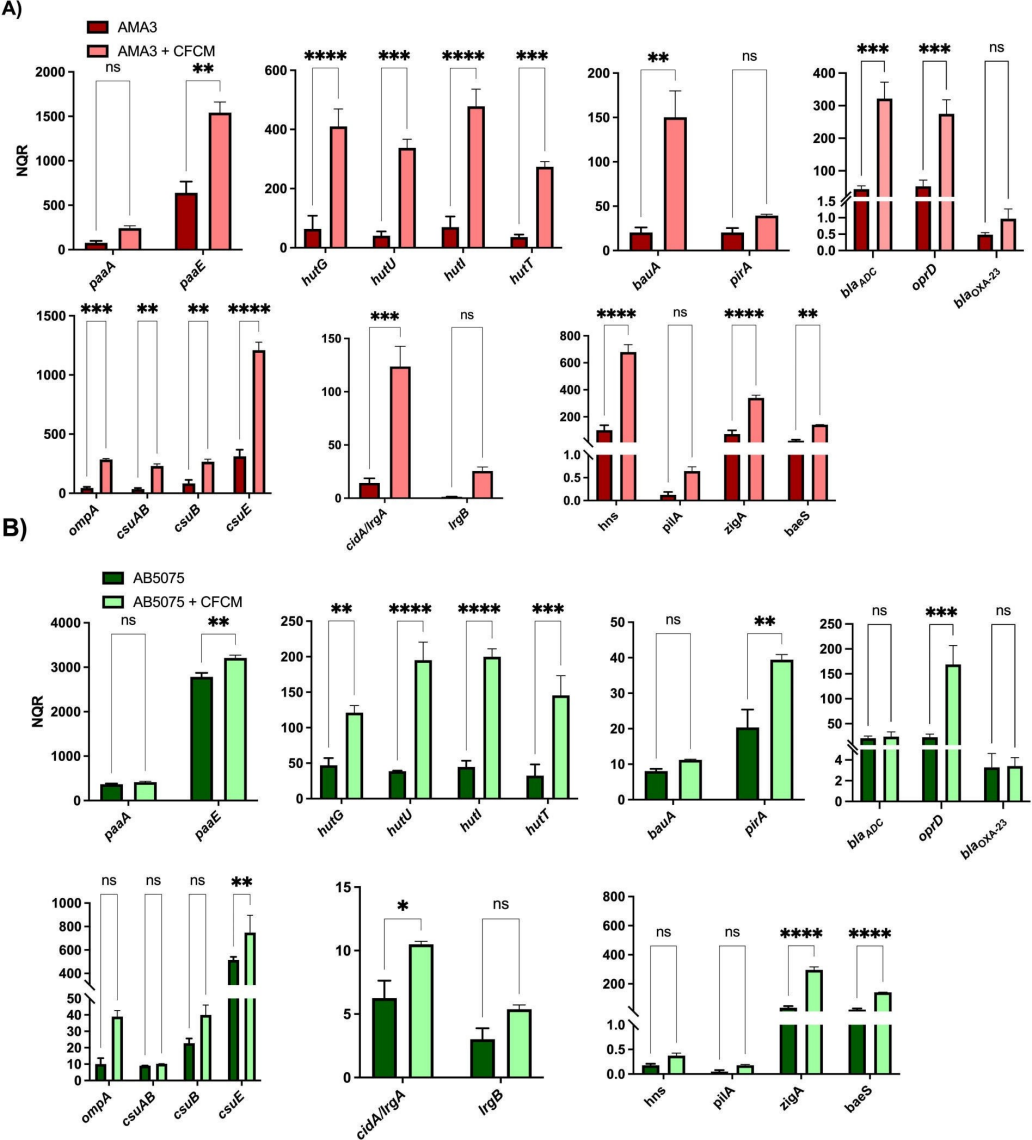
Transcriptional response of *A*. *baumannii* to *Lcb*. *rhamnosus* CRL 2244 CFCM. The clinical isolates AMA3 (A) and AB5075 (B) were grown with and without sub-MIC concentration of lyophilized *Lcb*. *rhamnosus* CRL 2244 CFCM for 18 h at 37°C. The differential genes expression was determined by qRT-PCR of genes involved in the phenylacetic acid catabolic pathway (*paaA* and *paaE*), histidine (*hutG*, *hutU*, *hutI* and *hutT*) and iron metabolism (*bauA* and *pirA*), genes associated with antibiotic resistance (*ampC*, *oprD* and *bla*_OXA-23_), genes related to biofilm formation (*ompA*, *csuAB*, *csuB* and *csuB*), genes involved in programmed cell death (*IrgA/cirA* and *IrgB*), and genes associated with bacterial virulence, including those related to zinc metabolism (*zigA*), pili production (*pilA*), efflux functions (*baeS*), and the global transcriptional regulator *hns*. The data presented are the mean ± standard deviation (SD) of normalized relative quantities (NRQ) derived from transcript levels calculated using the qBASE method. Statistical significance (*P* < 0.05) was determined by one-way ANOVA followed by Tukey’s multiple-comparison test. **, *P* < 0.01; ***, *P* < 0.001; ****, *P* < 0.0001; ns, not significant. Technical triplicates using three independent biological samples were used to collect experimental data. Error bars represents the standard deviation (SD).

To explore how *A*. *baumannii* transcriptionally responds when exposed to lyophilized CFCM, we conducted quantitative RT-PCR (qRT-PCR) assays using the AMA3 (Fig 4A) and AB5075 (Fig 4B) strains. Both strains showed a significant overexpression of genes associated with *A*. *baumannii* virulence, including those involved in phenylacetate (PAA) catabolism, such as *paaA*, *paaE*; and the *hutG*, *hutU*, *hutI*, *hutT* genes, all of which are components of the histidine catabolic Hut pathway described in other bacteria (Fig 4). Furthermore, a significant increase in the expression of additional genes involved in the virulence of *A*. *baumannii* was observed, including the iron metabolism-related genes *bauA* and *pirA* genes in AMA3 and AB5075 respectively. Genes associated with biofilm formation such as *ompA*, *csuAB*, *csuB* and *csuE* were significantly upregulated in AMA3, while only *csuE* was seen significantly increased in AB5075 (Fig 4). In addition, genes associated with antibiotic resistance were also studied; *oprD* was significantly increased in both strains, while *bla*_ADC_ was increased only in AMA3 (Fig 4). We also noted the significant upregulation of genes related to zinc metabolism (*zigA*) and efflux functions (*baeS*) in both strains; however, the transcriptional expression of the *hns* global regulator was significantly increased only in AMA3 (Fig 4). Interestingly, the gene encoding the CidA/LgrA protein, which is involved in programmed cell death in some bacteria, was also found to be significantly overexpressed in both strains (Fig 4).

Taken together, these results indicate that the presence of *Lcb*. *rhamnosus* CRL 2244 CFCM in the extracellular medium results in the differential expression of genes associated with a variety of cellular functions such as cell metabolism, host pathogen-interactions and even bacterial death. However, this transcriptional response varies between AB5075 and AMA3, with the latter strain displaying a higher number of differentially transcribed genes.

## Discussion

The lack of progress in the development of novel and effective antibiotics against MDR pathogens such as CRAB strains underscores the need for research into innovative approaches to address bacterial infections. Consequently, ongoing research efforts are focused on introducing therapeutic alternatives capable of slowing the evolution of antimicrobial resistance [26]. Probiotic LAB constitute a natural and safe therapeutic strategy. The antimicrobial activity of specific LAB strains or their extracellular products against pathogens is well documented in the literature [10, 12, 15]. LAB can produce numerous extracellular antimicrobial compounds because of their primary and secondary metabolisms, which can exert an antagonistic or antimicrobial action. These include the production of organic acids (mainly lactic and acetic acid), with a concomitant reduction in pH that limits the growth of other microorganisms [12, 27]; the production of other antimicrobial substances such as ethanol, diacetyl, hydrogen peroxide, reuterin and bacteriocins, among others [13, 27–30].

Nevertheless, limited research has been conducted on the potential use of LAB against extremely resistant strains such as CRAB clinical isolates [10, 13]. Published studies have indicated the antagonistic effects of different LAB against *A*. *baumannii*; however, there remains a need to identify the specific effectors and mechanisms responsible for their biological actions [10–13, 15]. Previously, in co-culture conditions, we observed a potent lethal activity of *Lcb*. *rhamnosus* CRL 2244 on *A*. *baumannii* strains [15]. Also, the transcriptomic analysis of *A*. *baumannii* cells co-cultured with *Lcb*. *rhamnosus* CRL 2244, revealed the differential expression of several genes mainly associated with metabolic pathways [15]. Our results indicated that *A*. *baumannii* actively responds to the stress imposed by LAB to enhance its survival strategies [15]. Based on these preliminary and published observations, we further evaluated in this present study whether the secretion of a substance(s) by *Lcb*. *rhamnosus* CRL2244 has the potential to induce or contribute to the death of CRAB cells as well as other MDR pathogens. Our findings revealed that the CFCM obtained after 96-h incubation presents a potent antimicrobial activity, with greater efficacy against Gram-negative bacilli, particularly CRAB strains. The transwell assay validated the antimicrobial effect observed with CFCM, indicating the presence of a compound(s) in the culture medium responsible for the killing activity. Notably, variations in pH, temperature, and exposure to different enzymes did not have a significant impact on the antimicrobial activity. Ladha *et al*. (2020) [11] identified a natural low molecular weight compound, containing a piperazine ring, produced by *L*. *plantarum* LJR13. This compound can inhibit the growth of *A*. *baumannii*, *Listeria monocytogenes* and *S*. *aureus* by forming vesicles and pores in the cell wall of sensitive cells. Other authors identified a common compound in the supernatant of twenty LAB strains, including species such as *L*. *plantarum*, *L*. *paraplantarum*, *Pediococcus pentosaceus*, *P*. *acidilactici*, *Pediococcus* spp., and *Enterococcus* spp. This compound, belonging to the diketopiperazine group, proved to be an effective agent against extensively drug-resistant *A*. *baumannii* [13]. Although the structure of the compound(s) secreted by *Lcb*. *rhamnosus* CRL 2244 remains unknown, the results presented in here suggest that it is a low molecular weight non-peptide organic compound that is actively stable after being exposed to changes in temperature, pH and enzymatic treatment.

Interestingly, the compound produced by *Lcb*. *rhamnosus* CRL2244 exhibits a synergistic interaction with the β-lactam antibiotic meropenem, restoring susceptibility to it in carbapenem-resistant strains. The synergistic interaction between natural compounds or other bioactive components, such as biogenic metallic nanoparticles, and existing antibiotics proves to be an effective approach in combating the resistance phenomenon, as they can facilitate the interaction between an antimicrobial agent and its target within the pathogen [21, 31–33]. Several authors have documented synergistic effects between natural products and antibiotics from different classes, employing diverse mechanisms against various MDR pathogens [21, 33–35]. However, understanding the mode of action of the *Lcb*. *rhamnosus* CRL2244 secreted compound(s) and its synergistic interaction with clinically relevant antibiotics requires further investigation. Nevertheless, we hypothesize that influencing the integrity of the cell membrane, as suggested by morphological changes observed in SEM images, such as elongation of cell length and a smoother bacterial surface appearance, is one of the mechanisms by which the *Lcb*. *rhamnosus* CRL2244 exerts it antibacterial activity.

The analysis of the transcriptional response *A*. *baumannii* exposed to a sub-MIC concentration of the CFCM from *Lcb*. *rhamnosus* CRL 2244 revealed an overexpression of genes coding for critical metabolic functions. These functions include the acquisition of the essential metals Zn and Fe, as well as the expression of key metabolic pathways, including the PAA catabolic pathway and the expression of the Hut system. It is well documented that the *paa* operon is one of the most differentially regulated *A*. *baumannii* pathways in response to numerous stress conditions, including the presence of antibiotics, and its role in antibiotic resistance; whereas the Hut histidine catabolic pathway provides carbon, nitrogen energy during infection [36, 37].

Another noteworthy finding is the significant overexpression of the *cidA/lrgA* genes, also consistent with our previous observations collected using a co-culture approach [15]. These genes are linked to programmed cell death in other bacteria, potentially through holin-antiholin activity or the regulation of cell death during overflow metabolism [38, 39]. Considering all these observations, we can speculate that the compound(s) produced and secreted by *Lcb*. *rhamnosus* CRL2244 modulate the *A*. *baumannii* transcriptional response to combat stress signals, which ultimately can affect cell integrity.

## Conclusions

The present work revealed *Lcb*. *rhamnosus* CRL2244 releases a small compound(s) that has bactericidal activity against different MDR pathogens. In addition, a decrease of MIC values against selected MDR strains was detected when CFCM samples were combined with meropenem. We hypothesize that this response could be due to changes in cell morphology, the differential expression of genes coding for critical metabolic pathways and the expression of *A*. *baumannii* virulence functions including biofilm formation. Future experiments to further characterize and identify the nature and chemical structure of the released compounds(s) are needed. In sum, the results presented in this report, suggest the potential use of *Lcb*. *rhamnosus* CRL2244 released compound(s) as alternative and/or complementary options to treat infections caused by multi-drug resistant bacterial pathogens.

## Supporting information

S1 Table Antimicrobial activity of *Lacticaseibacillus rhamnosus* CRL 2244 extracts. (DOCX)

S1 Fig Representative pictures showing the antimicrobial activity of CFCM samples obtained from 48-h, 96-h and 120-h cultures on multidrug pathogens tested by spot-on-the-lawn method. (TIF)

S2 Fig Representative pictures of the synergistic activity of lyophilized CFCM resuspended in PBS of *Lacticaseibacillus rhamnosus* CRL 2244 with antibiotics on CRAB and MRSA strains. The assays were performed in three independent technical and biological samples.
(TIF)

S3 Fig Macro-colony assays showing the effect of lyophilized CFCM resuspended in PBS on PNAG production. Bacterial cultures were gown on CAMHA supplemented with 2% glycerol and 40 μg/mL of Congo red. This is a representative image of assays performed in technical and biological triplicates.
(TIF)
