# Supplementary material for: Lacticaseibacillus rhamnosus CRL 2244 secreted metabolites display killing and antibiotic synergistic activity against multi-drug resistant pathogens
Source: PLoS One. 2024 Jun 28;19(6):e0306273. doi: 10.1371/journal.pone.0306273 (PMC11213291; doi:10.1371/journal.pone.0306273)
Supplement: S1 Table — (DOCX) [file pone.0306273.s001.docx]

**S1 Table. Antimicrobial activity of *Lacticaseibacillus rhamnosus* CRL 2244 extracts.**

| **Inhibition halo diameters (IDH mm)** | | | | |
| --- | --- | --- | --- | --- |
| **Acetone precipitation** | | | | |
| **Strain** | Aqueous | Organic | DMSO | CFCM-96^a^ |
| **AB5075** | 14 | 20 | 0 | 20 |
| **AMA3** | 12 | 24 | 0 | 20 |
| **Ethyl acetate extraction** | | | | |
| **Strain** | Aqueous | Organic | DMSO | CFCM-96 |
| **AB5075** | 8 | 20 | 0 | 20 |
| **AMA3** | 20 | 22 | 0 | 20 |

^a^Used as control
