# Supplementary figures and images for: Lacticaseibacillus rhamnosus CRL 2244 secreted metabolites display killing and antibiotic synergistic activity against multi-drug resistant pathogens
Source: PLoS One. 2024 Jun 28;19(6):e0306273. doi: 10.1371/journal.pone.0306273 (PMC11213291; doi:10.1371/journal.pone.0306273)

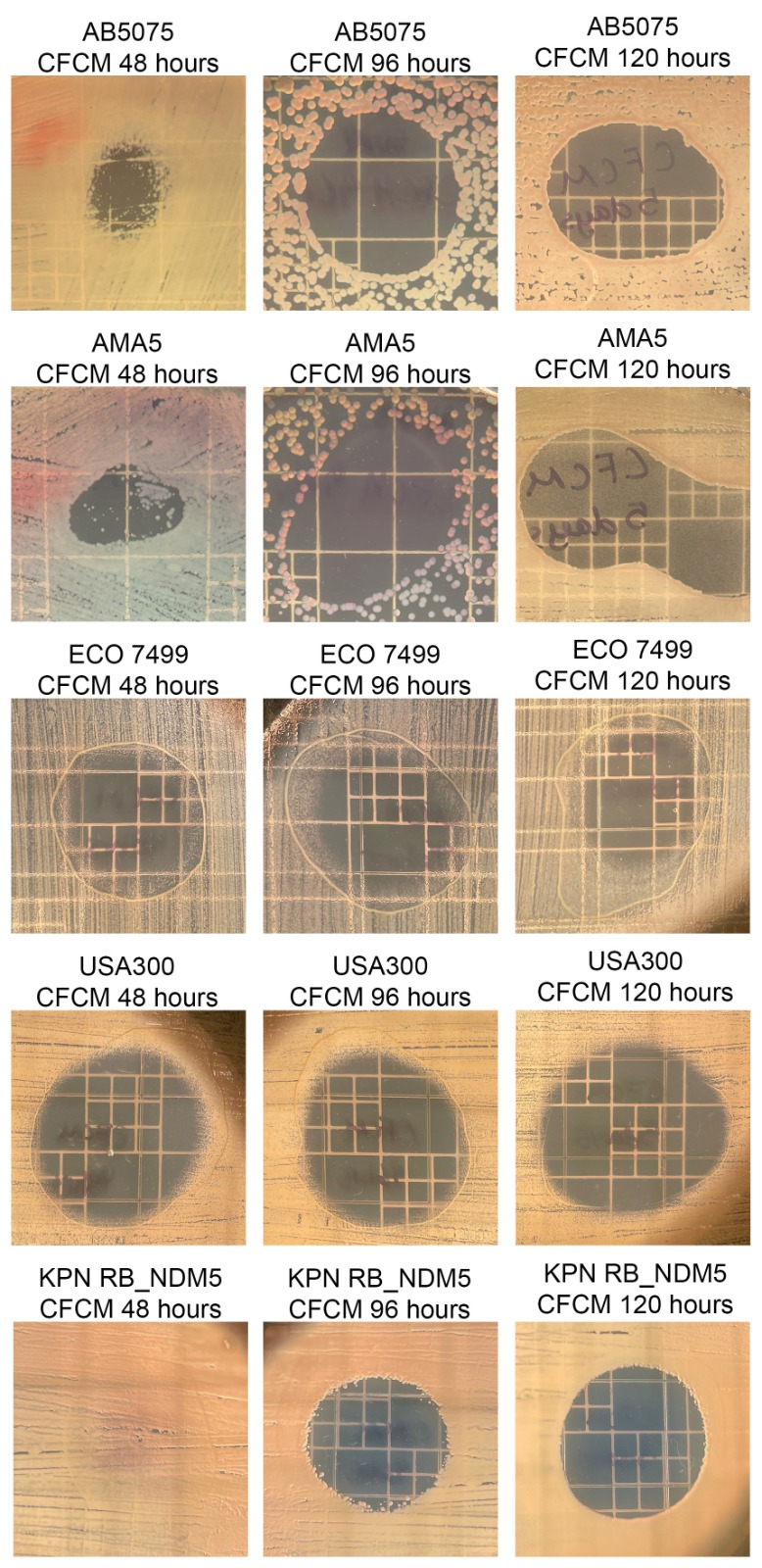

Supplement: S1 Fig — (TIF) [file pone.0306273.s002.tif]

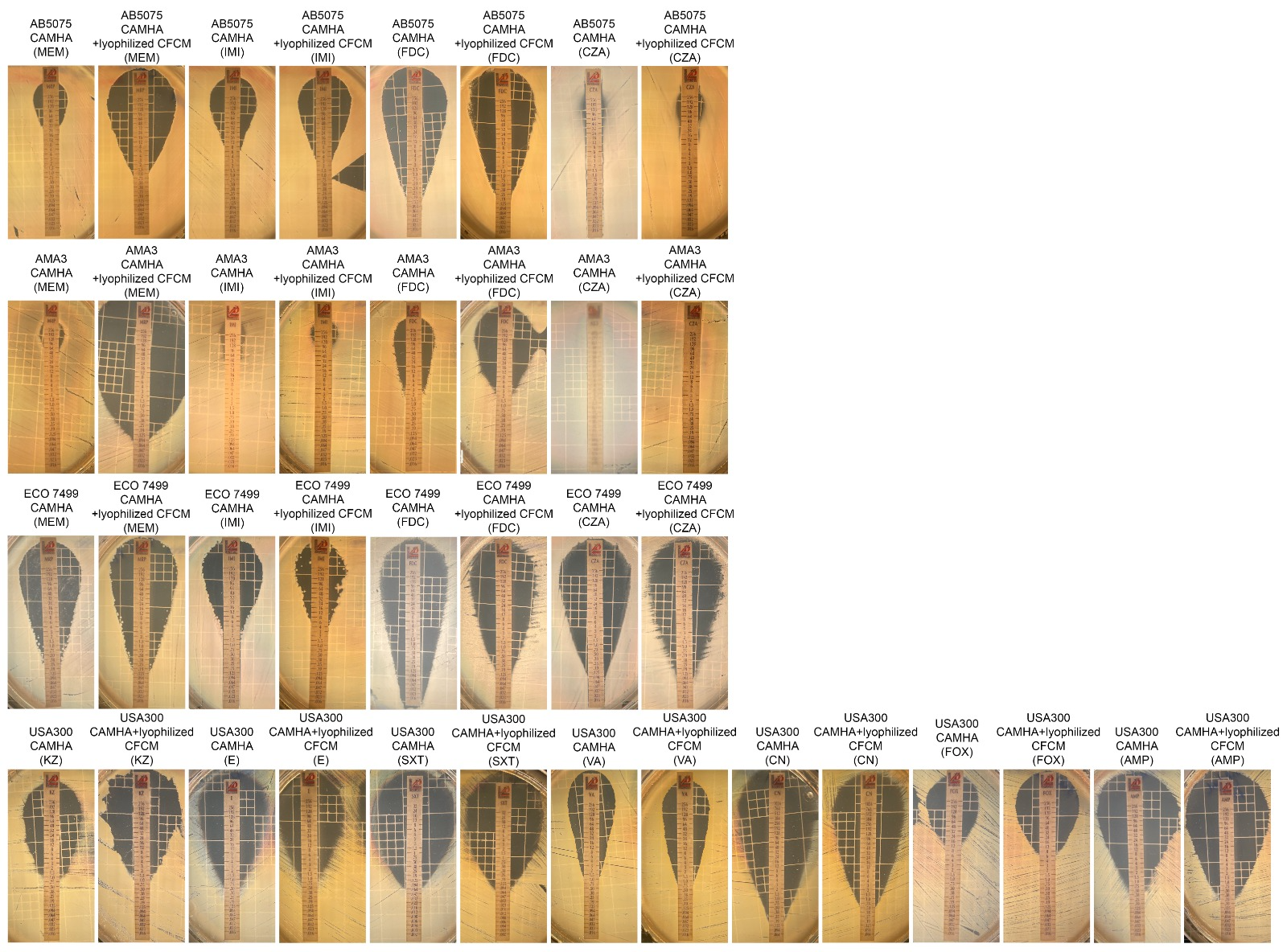

Supplement: S2 Fig — The assays were performed in three independent technical and biological samples. (TIF) [file pone.0306273.s003.tif]

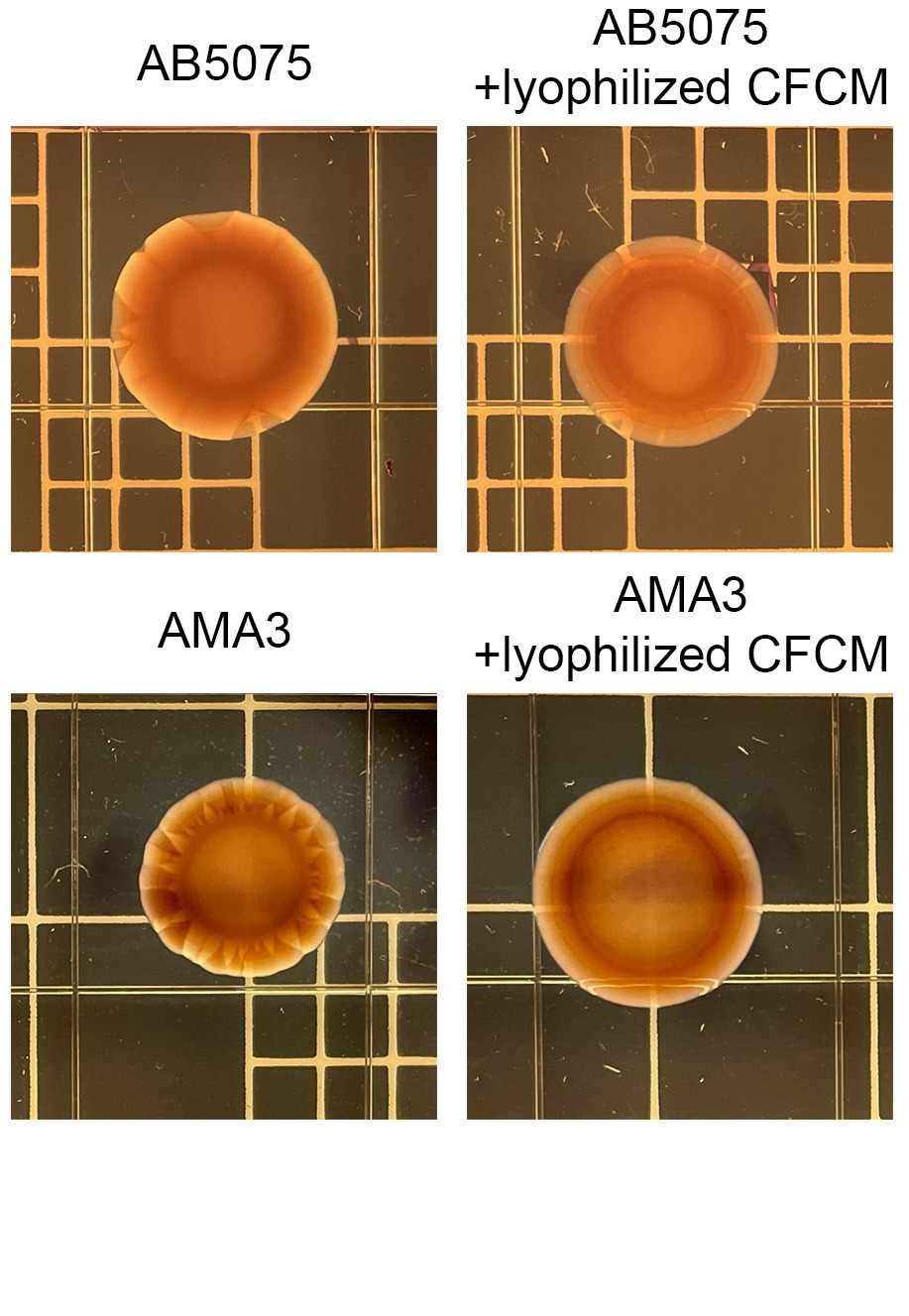

Supplement: S3 Fig — Bacterial cultures were gown on CAMHA supplemented with 2% glycerol and 40 μg/mL of Congo red. This is a representative image of assays performed in technical and biological triplicates. (TIF) [file pone.0306273.s004.tif]
